# Microarray based transcriptome profile data of ∆*lon* and ∆*lon rpoB12* strains of *Escherichia coli*

**DOI:** 10.1016/j.dib.2018.10.020

**Published:** 2018-10-09

**Authors:** Shanmugaraja Meenakshi, M. Hussain Munavar

**Affiliations:** Department of Molecular Biology, School of Biological Sciences, Centre for Advanced Studies in Functional and Organismal Genomics, Madurai Kamaraj University [University with Potential for Excellence], Madurai 625021, India

**Keywords:** ∆*lon*, *rpoB12*, Capsule, Biofilm, Motility, Stress response

## Abstract

The data presented in this article shows the microarray based transcriptome profiles of ∆*lon* and ∆*lon rpoB12* strains of *Escherichia coli*. The *rif* mutation namely, *rpoB12* was isolated spontaneously in the background of ∆*lon* strain (over-produces colanic acid capsular polysaccharide) as a suppressor for over-production of colanic acid capsular polysaccharide (Meenakshi and Munavar, 2015) [1]. The *E. coli* strains were grown in LB medium at 30 °C overnight in duplicates. Total RNA from each samples were isolated and microarray based transcriptome profiles were studied and compared. The detailed methodology and data are given in this article. The interpretation of these data are discussed in the research article, “Evidence for Up and Down Regulation of 450 genes by *rpoB12* (*rif*) Mutation and their Implications in Complexity of Transcription Modulation in *Escherichia coli*” (Meenakshi and Munavar, 2018) [2].

## Specifications table

**Table:** 

Subject area	*Biology*
More specificsubject area	*Microbial Molecular Biology*
Type of data	*Table, transcriptome data*
How data was acquired	*The data from the images were extracted using Agilent Feature Extraction software version 11.5.1.1 and the extracted raw data was analyzed using Agilent GeneSpring GX version 12.0. The data were normalized using 75th percentile shift in comparison with the control sample.*
Data format	*Analyzed data.*
Experimental factors	*Escherichia coli strains namely SG20780 (∆lon) and MMR6 (∆lon rpoB12) are used as samples.*
Experimental features	*Above said E.coli strains were grown in LB for overnight and sub-cultured to attain mid-log phase at 30 °C. Total RNA was isolated and subjected for Microarray based transcriptome analysis.*
Data source location	*Tamil Nadu, India.*
Data accessibility	*Data are available in this article*
Related research article	*“Evidence for Up and Down Regulation of 450 genes by rpoB12 (rif) Mutation and their Implications in Complexity of Transcription Modulation in Escherichia coli”* [2].

## Value of data

• This is perhaps the first report showing the genome-wide expression of a *rif* (*rpoB*) mutant of *E. coli*. The data clearly gives explanation for the pleiotropic phenotype associated with *rpoB12* (His526Tyr) mutation that are reported earlier.
• Data reported herein shows that a certain group of genes are getting up regulated and certain other group of genes are getting down regulated while the expression of many a genes are unaltered. This observation portrays a vital role for the specific promoter elements/its associated factors along with the fast moving RNA polymerase, which in combination might result in modulation of expression of genes.
• The *rpoB12* mutation reported herein has already been reported by many research groups in different contexts more specifically, His426Tyr change has been reported in higher frequency in Mycobacterium when exposed to Rifampicin [3], [4], [5]. Therefore, the data presented here gives clue for the association of this *rif* mutation with virulence and Rif resistance.
• Since *E. coli* and other pathogenic microbes such as *Salmonella*, *Pseudomonas*, *Mycobacteria*, *Erwinia etc.*, share higher percentage of homology [6], [7], [8], this data would be immensely helpful to understand pathogenesis and antimicrobial resistance in these pathogenic microbes.

## 1 Data

The *rif* mutation namely, *rpoB12* is identical to the previously reported *rif* mutation, *rpoB2* [3]. This mutation is reported to be associated with pleiotropic phenotype in *E. coli* [1], [9]. Since *rpoB12* mutation (His526Tyr) was found to be clinically relevant, microarray based transcriptome profile was studied in this *rif* mutant. The analysed data of the above said experiment is presented in this article (Table 1).

**Table 1:** Summary of the microarray data analyses.

Name of the strains	Sample	No. of. Genes up regulated	No. of. Genes down regulated
SG20780	Control	NA	NA
SG2078_R	Control (Replicate)		
MMR6	Test	753	643
MMR6_R	Test (Replicate)		

## 2 Experimental design, materials and methods

### 2.1 Growth conditions of bacterial cultures

Overnight grown cultures of SG20780 (∆*lon cps-lac*) and MMR6 (∆*lon cps-lac rpoB12*) were sub-cultured in duplicates and the mid-log phase cultures were used for the microarray experiment. Refer [2] for more details.

### 2.2 RNA extraction

Given below is the brief summary of methods followed by the company; Isolation of RNA from *E. coli* was carried out by using combination of Trizol and Qiagen RNeasy mini kit with DNase treatment (Cat # 74106).

### 2.3 RNA quality control

The concentration and purity of the RNA were evaluated using the Nanodrop Spectrophotometer (Thermo Scientific; 1000). The value of Abs260/280 for the samples ranged from 1.8 to 2. The value of Abs260/230 ranged from 1.5 to 1.8. The integrity of the RNA samples were analysed on the Bioanalyzer (Agilent; 2100). The RNA integrity number of the samples ranged from 8 to 10.

### 2.4 Labelling

The samples for Gene expression were labelled using Agilent Quick-Amp labelling Kit (p/n5190-0442). 500 ng each of total RNA were reverse transcribed at 40 °C using primer mix of random hexamer and oligo-dT primer tagged to a T7 polymerase promoter and converted to double stranded cDNA. Synthesized double stranded cDNA molecules were used as template for cRNA generation. cRNA was generated by *in vitro* transcription and the dye Cy3 CTP(Agilent) was incorporated during this step. The cDNA synthesis and *in vitro* transcription steps were carried out at 40 °C. Labelled cRNA was cleaned up using Qiagen RNeasy columns (Qiagen, Cat no.: 74106) and quality assessed for yields and specific activity using the Nanodrop ND-1000.

### 2.5 Hybridization and scanning

1000 ng of labelled cRNA sample were fragmented at 60 °C and hybridized on to a Genotypic designed *E coli*_8 × 15K (AMADID: 020304). Fragmentation of labelled cRNA and hybridization were done using the Gene Expression Hybridization kit (Agilent Technologies, *in situ* Hybridization kit, Part no. 5190-0404). Hybridization was carried out in Agilent׳s Surehyb Chambers at 65 °C for 16 h. The hybridized slides were washed using Agilent Gene Expression wash buffers (Agilent Technologies, Part no. 5188-5327) and scanned using the Agilent Microarray Scanner (Agilent Technologies, Part no. G2600D).

### 2.6 Feature extraction

Data extraction from Images was done using Feature Extraction software Version 11.5.1.1 of Agilent.

### 2.7 Microarray data analysis

Feature extracted raw data was analyzed using GeneSpring GX Version 12.0 software from Agilent. Normalization of the data was done in GeneSpring GX using the 75th percentile shift and normalized to Specific control Samples (Percentile shift normalization is a global normalization, where the locations of all the spot intensities in an array are adjusted. This normalization takes each column in an experiment independently, and computes the *n*th percentile of the expression values for this array, across all spots (where *n* has a range from 0 to 100 and *n* = 75 is the median, it subtracts this value from the expression value of each entity)). Significant genes up and down regulated in the test samples with respect to control sample were identified. Statistical *T*-test *p*-value was calculated based on volcano Plot. Differentially regulated genes were clustered using hierarchical clustering based on Pearson coefficient correlation algorithm to identify significant gene expression patterns among the three different conditions. Genes were also classified based on functional category and pathways using DAVID database tool.

## Appendix A Supplementary material

The analysed data of the differentially regulated genes in MS Excel Format.
